# Thoracoscopic Assisted PleuralPort^TM^ Application in Seven Dogs Affected by Chronic Pleural Effusion

**DOI:** 10.3390/vetsci10050324

**Published:** 2023-04-29

**Authors:** Amanda Bianchi, Francesco Collivignarelli, Andrea Paolini, Massimo Vignoli, Gert W. Niebauer, Giulia Dolce, Sara Canal, Andrea De Bonis, Martina Rosto, Francesca Del Signore, Roberto Tamburro

**Affiliations:** 1 Department of Veterinary Medicine, University of Teramo, 64100 Teramo, Italy; fcollivignarelli@unite.it (F.C.); mvignoli@unite.it (M.V.); gertnieb@tin.it (G.W.N.); scanal@unite.it (S.C.); adebonis@unite.it (A.D.B.); martina_rosto@hotmail.it (M.R.); fdelsignore@unite.it (F.D.S.);; 2 Roma Sud Veterinary Clinic, Via Pilade Mazza, 00173 Roma, Italy; gveteunipg@gmail.com

**Keywords:** PleuralPort^TM^, pleural effusion, dog, oncology, chylothorax, mesothelioma, intracavitary chemotherapy

## Abstract

**Simple Summary:**

The aim of the study is to describe the application, complications, and outcome of thoracoscopic PleuralPort^TM^ in dogs affected by chronic pleural effusion. Seven dogs received eight devices for the management of five effusions caused by malignant mesothelioma, one caused by pleural carcinomatosis, and pulmonary metastasis, and one chronic chylothorax. One dog developed pneumothorax that was resolved after 12 h, and, in another, a tube was obstructed 45 days post application. The median duration of the port placement in the four cancer patients was 5 months and all dogs were euthanized for disease progression; in the dog with chylothorax, the device was removed after 1 year at the resolution of effusion.

**Abstract:**

Chronic non-septic pleural effusion is a condition that frequently may occur because of lung or pleural neoplasia, or chylothorax refractory to surgical treatment, in dogs. Effusion management can be performed with multiple pleurocenteses or the application of chest drains. New modified vascular devices have been used for patients with chronic diseases; they offer the advantage of allowing home management and do not require hospitalization. Eight PleuralPort^TM^ devices were applied in seven dogs during thoracoscopic exploration and biopsy procedures; five were affected by mesothelioma; one by lung metastases from a mammary carcinoma; and one by chronic chylothorax. The median time of surgical procedure was 51 min; one developed pneumothorax post-operatively that resolved within 12 h after repeated drainage; one device was obstructed after 45 days and was successfully managed by flushing. All patients were discharged after 24 h. The median duration of port insertion in cancer patients was 5 months and those dogs were euthanized because of tumor progression; in the dog with chylothorax, the device was removed after 1 year when the effusion had resolved.

## 1. Introduction

Chronic non-septic pleural effusion is a pathological fluid accumulation within the pleural space, which, in small animals, is often the consequence of thoracic malignancies and/or is not responsive to surgical treatment, e.g., thoracic duct ligation for the treatment of chylothorax [1].

Pleural effusion can cause dyspnea, panting, weakness, and exercise intolerance, which may have a significant impact on the quality of life; if not treated, it can lead to pleural fibrosis and death [2]. The effectiveness of the treatment of pleural effusion depends on identifying and resolving the underlying cause, and, when not possible, palliative management aims to drain fluid from the pleural space to improve the clinical condition [3]. In order to treat such conditions, multiple thoracocenteses and the application of thoracostomy tubes can be used [4]. The two techniques are associated with several possible complications: iatrogenic damage (pneumothorax, hemorrhage) [5], procedure-induced infections, and drainage tube damage or dislodgement caused by the patient [6]. In both cases, the procedures must be performed in a hospital facility and, if drains are applied, the patients must be hospitalized [7].

Several surgical procedures have been described to treat or manage chronic pleural effusion. Pleuroperitoneal and pleurovenous shunts have limited use due to the many associated complications, such as obstruction, infection, suction pump ineffectiveness, and the potential risks of metastatic dissemination, thrombosis, and tumor embolization [1,8,9,10].

The use of devices such as the PleuralPort™ (Norfolk Vet Products, Skokie, IL, USA) has been described to manage this condition [11]; PleuralPort™ consists of a fenestrated tube that is inserted into the thoracic cavity connected to a perforable hub located in a subcutaneous pocket. Fluid is aspirated using a Huber needle inserted percutaneously into the hub after aseptic preparation of the area. The drainage can be performed at home by the owners. The application of the PleuralPort™ has been described percutaneously and during the thoracotomy procedure [11,12]. The latter, which is more invasive, however, allows the exploration of the thoracic cavity and the simultaneous performance of surgical biopsies. There are currently no reports describing the placement of a PleuralPort™ device in minimally invasive surgery [3]. A thoracoscopy allows for the exploration of the pleural cavity, aids to identify the underlying cause of the effusion, and offers the possibility of performing biopsies of the visible lesions [13]. A thoracoscopy results in less pain and faster postoperative recovery compared to traditional thoracotomy [14]. Additionally, it can be helpful to recognize the detailed surgical anatomy by the magnification of the operative field through the telescope [15].

The aim of this study was to evaluate the technique and feasibility to place PleuralPorts™ via thoracoscopy in canine clinical cases of chronic pleural effusion and describe the related complications and clinical outcomes.

## 2. Materials and Methods

The study was approved by the Scientific Ethics Committee of the University of Teramo Protocol Number 1296/2023. The owner’s written consent was obtained for every animal enrolled. All the dogs presented between 2019 and 2021, fulfilling the following inclusion criteria: chronic non-septic pleural effusion caused by malignancy or thoracic duct rupture (confirmed by cytology and cytochemical analysis). The exclusion criterion was a chronic pleural effusion secondary to cardiac or hepatic diseases. Each dog underwent the same diagnostic workup, including a complete blood count, serum biochemistry, cytology, and cytochemical analysis of the pleural fluid, and three-projection thoracic radiographs were performed. An exploratory thoracoscopy alone or in association with surgical biopsies were carried out in all the dogs. At the end of the procedure, the PleuralPort™ (Norfolk Vet Products, Skokie, IL, USA) devices were introduced. Signalment, history, diagnostic imaging, thoracic fluid analysis, the PleuralPort™ placement technique, surgical time, the duration of port function, the duration of port implantation, histopathological examination, complications, the chemotherapy protocol, and the clinical outcome were reported.

### 2.1. Surgical Procedure

Dogs received intravenously methadone (Semfortan^®^; Eurovet Animal Health, Bladel, The Netherlands) 0.3 mg/kg, propofol (Propovet^®^; Zoetis, Rome, Italy) to effect endotracheal intubation, and were maintained with isoflurane (IsoFlo^®^; Zoetis, Rome, Italy) in 100% oxygen. Intercostal blocks with 0.5 mL lidocaine 2% (Ecuphar SRL, Milan, Italy) were performed from the 8th to 12th rib. Intermittent positive pressure ventilation (IPPV) was set prior to the surgical procedure. The patients were positioned in the right or left lateral recumbency depending on the effusion and/or lesion site. The entire hemithorax from the scapula spine to the cranial abdomen and from the dorsal spine to the sternum was surgically prepared. The surgeon and surgeon’s assistant stood on the side of the patient, facing the dog’s affected hemithorax, and the endoscopic tower was placed on the contralateral side of the patient.

**Figure 1:**
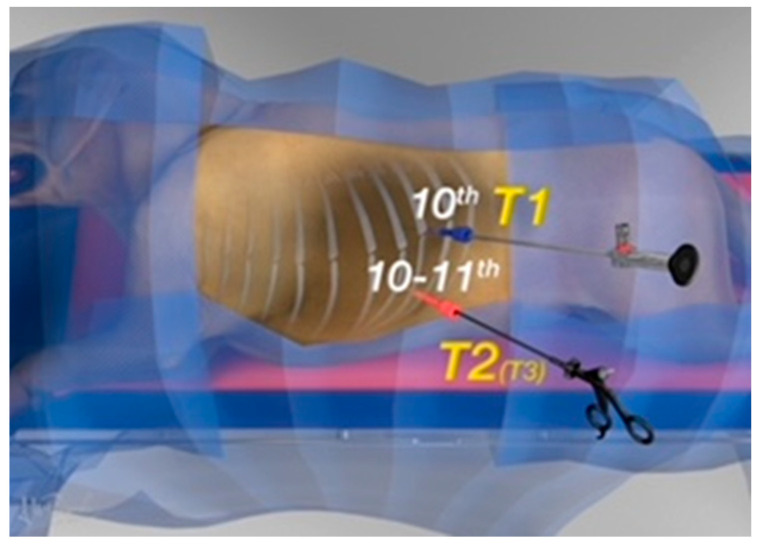
The illustration shows the thoracoscopic port placement. T1 was inserted in the dorsal third of 10th intercostal space, T2 between the middle third of the 10−11th intercostal space.

A 1 cm skin incision was made using a #11 scalpel blade in the dorsal third of the 10th intercostal space, for the insertion of a 6 mm cannula (62160GBK, Karl Storz endoscopy, Verona, Italy) (T1 in Figure 1). The 5 mm telescope (Hopkins^®^ II 62046 BA, Karl Storz endoscopy, Verona, Italy) was then introduced, and an exploratory thoracoscopy was performed. Under direct visualization, the second port (62160GBK, Karl Storz endoscopy, Verona, Italy) was created in the middle third of the 10–11th intercostal space (T2 in Figure 1).

After the port placement, an inspection of the hemithorax was performed to confirm the diagnosis and any lesions on the pleural surface or pulmonary parenchyma were biopsied with endoscopic biopsy forceps (66321DB, Karl Storz endoscopy, Verona, Italy).

**Figure 2:**
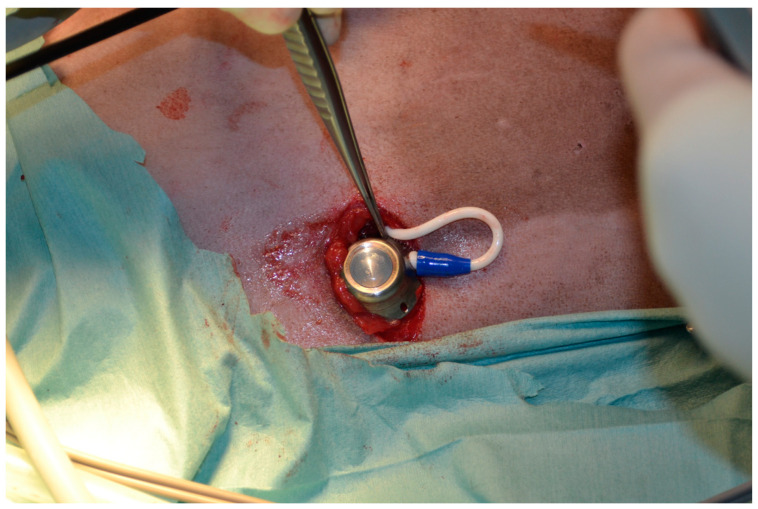
The location of the hub in the subcutaneous pocket after PleuralPort^TM^ placement.

A semicircular skin incision was made with an Airplasma^®^ (Airplasma^®^, Onemytis^®^, Alessandria, Italy) device, larger than the port on the pre-determined area (around the 10th intercostal space). A flap was created with Airplasma^®^ by dissection of the subcutaneous tissue [16,17]. The fenestrated part of the pleural drainage was advanced into the cranial portion of the hemithorax, taking care not to injure the lung parenchyma and avoiding crossing the mediastinum. The non-fenestrated part of the tube was tunneled subcutaneously and connected with a hub located in the subcutaneous pocket previously created in the caudal thorax (Figure 2).

**Figure 3:**
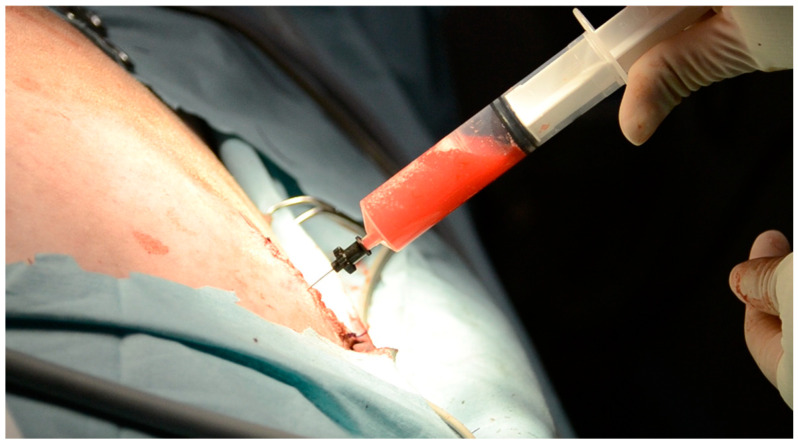
A Huber needle inserted in the subcutaneous hub at the end of the surgical procedure to assess the port’s function.

The PleuralPort^TM^ was transfixed to the thoracic wall (the fascia of the latissimus dorsi) with monofilament non-absorbable interrupted sutures (3-0 polypropylene) passed through hub holes. The system’s function was assessed with a Huber needle connected to an extension set by aspiration (Figure 3). 

The port was covered with the skin flap, which was then routinely closed.

### 2.2. Post-Operative Management

**Figure 4:**
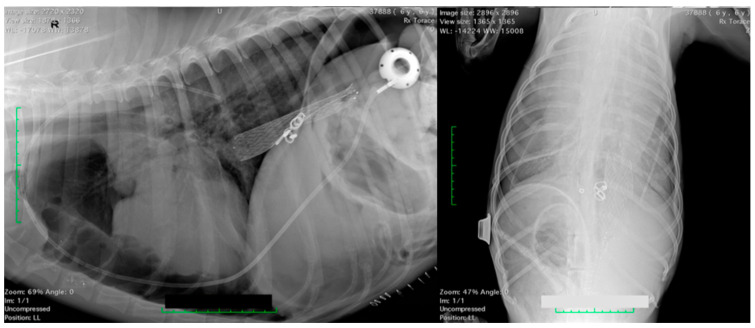
Case 3: a radiographic study of the thorax (right lateral and ventrodorsal), after PleuralPort^TM^ application. This dog had a caval stent placed 3 years before.

A radiographic study was performed to verify the correct tube position (Figure 4).

The initial frequency of aspiration from the drain was every 12–24 h, based on clinical signs and ultrasound evaluation of the thorax quadrants, and, before every aspiration, the skin was aseptically prepared with a 0.05% chlorhexidine solution and the tube was flushed with a sterile isotonic saline solution. 

Post-operative pain was assessed with the Italian Version of the Glasgow Compositive Measure Pain Scale-Short Form (ICMPS-SF) [18]. In the case of a pain score >5/24, a rescue dose of Methadone 0.2 mg/kg was administered intramuscularly (IM). The owners were instructed on the use of the PleuralPort^TM^ device, with emphasis on aseptic skin preparation, the frequency of aspiration, and how to carry it out. These owner-performed manipulations were prescribed two times per day for the first week, then, according to clinical signs, once a day. In the case of exercise intolerance and dyspnea, aspiration through the hub was recommended.

### 2.3. Complications

Short-term complications were defined as complications that occurred intraoperatively or during the hospitalization period, such as pneumothorax, hemorrhage, fluid leakage from the insertion site, seroma, subcutaneous emphysema, and death.

Long-term complications were those observed during the entire period of implant insertion after discharge, such as surgical site infection, pyothorax, device obstruction, device displacement or migration, death.

### 2.4. Follow-Up Information 

Seven days post discharge, a clinical on-site evaluation was carried out, and telephone follow-up with owners or referring veterinarians was performed every week as long as the device remained in place, in order to assess the clinical response of patients and the management and functionality of ports.

### 2.5. Descriptive Statistics

The data distribution was assessed for normality with the Shapiro–Wilk test. Normally distributed data were presented as the mean and standard deviation, not normally distributed data were presented as median and range. The age, weight, surgery time, hospitalization time, and implant duration were presented as the median and range.

## 3. Results

Eight 9 French PleuralPort^TM^ devices were used in seven dogs with pleural effusion. 

The cohort included four neutered females and three males (two entire, one neutered). The breeds were Yorkshire (*n* = 2), German Shepherd (*n* = 1), Italian Greyhound (*n* = 1), Dobermann Pinscher (*n* = 1), Dachshund (*n* = 1), and mixed breed (*n* = 1). The median body weight was 5 kg (range 3–32); the median age was 11 years old (range 8–15).

The median surgical time, which included the pleural cavity exploration and biopsy, was 51 min (range 35–80). The average hospitalization time after port placement was 24 h (12–72) (Table 1).

**Figure 5:**
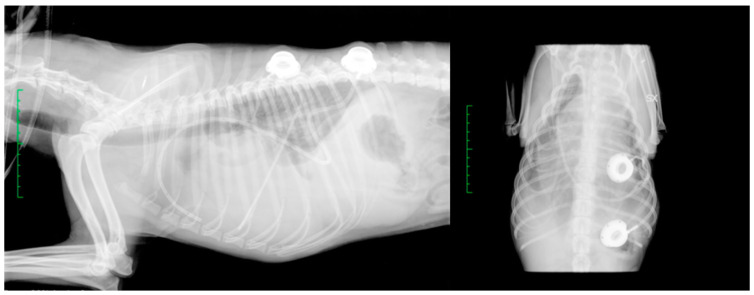
Case 1: two pleural ports were placed to manage a severe bilateral effusion due to malignant mesothelioma. Both the drains had subcutaneous hubs in the left hemithorax. The right drain was inserted into the chest by subcutaneous tunneling.

In five out of seven dogs, the pleural effusion was caused by malignant mesothelioma, in one of these dogs two PleuralPort^TM^ devices were applied because of bilateral pleural effusion (Figure 5).

In one dog, the pleural effusion resulted from pleural carcinomatosis and the presence of a pulmonary metastasis secondary to a mammary carcinoma. In one dog, the pleural effusion was a chronic chylothorax unresponsive to thoracic duct ligation performed 1 year before. 

The five dogs with pleural effusion because of malignant mesothelioma, in addition to regular aspiration, also received intracavitary chemotherapy as described by Moore et al. directly through the PleuralPort^TM^ devices [19]. In the other two dogs, the pleural ports were only used to manage pleural effusion by direct aspiration by the owner.

The five dogs with malignant mesothelioma (cases 1, 2, 3, 4, 5) received daily piroxicam at the dose of 0.3 mg/kg PO for 4 months and cisplatin at the dose of 50 mg/m^2^ every 3 weeks, intracavitary, directly through a PleuralPort^TM^, for a total of four doses.

### 3.1. Complications

A short-term complication was observed in one dog (case 7): pneumothorax developed after fluid aspiration. The condition resolved after 12 h by PleuralPort^TM^ aspiration every 3 h.

A long-term complication was observed in one dog (case 6). Forty-five days after application, the owners were unable to aspirate the fluid because of a tube obstruction. The dog was brought to the hospital and the tube was flushed with sterile isotonic saline solution. No other complications were seen.

### 3.2. Follow-Up Information 

During clinical follow-up at 7 days post-surgery, no patients showed signs of a surgical site infection, oedema, seroma, or fluid leakage around the device. The findings of the clinical examination were within the normal limits. 

In the dog with idiopathic chylothorax (case 7), chylous effusion resolved spontaneously after 1 year and the port was removed. Case 6 with pleural carcinomatosis due to pulmonary metastasis developed increasing volumes of effusion, fever, and episodes of collapse 5 months after port placement, and euthanasia was chosen. 

All the dogs affected by malignant mesothelioma were euthanized on average 5 months after the placement of the devices for disease progression (Table 1). The dogs affected by neoplasms with a recurrence of pleural effusion or increased fluid volume were subjected to euthanasia when there was a decline in quality of life with clinical signs such as anorexia, depression of the sensorium, weight loss, and generalized weakness. Disease progression in dogs with mesothelioma was identified by a ≥25% increase in efflux production compared to baseline [20].

## 4. Discussion

The study demonstrated the feasibility of a thoracoscopic PleuralPort^TM^ application in seven dogs with chronic pleural effusion. This condition is clinically challenging as the severity of clinical signs associated with the poor quality of life of the affected animals requires effective, often palliative, management [4]. When the underlying cause cannot be resolved, solutions have to be implemented to remove the fluid from the pleural cavity. Animals with chronic pleural effusion have a thickening of the pleura and possibly trapped lung lobes. Therefore, repeated thoracentesis can cause pneumothorax, as well as pyothorax [5].

Pleuroperitoneal, pleurovenous shunt and omentalization of the pleural space have been employed to manage chronic effusions, but they had limited success for the numerous complications described. In 2011, Brooks and colleagues described the use of the PleuralPort^TM^ device for the management of pleural effusion in six dogs and four cats [11]. In this case series, nine animals were affected by chylous effusion and one dog had pleural carcinomatosis. Eleven ports were placed, with one cat receiving bilateral ports. Complications were observed in four cases and, in particular, one cat was immediately subjected to euthanasia after implantation as consequence of pneumothorax. Port occlusion was observed in two dogs and one cat. Excluding the cat with pneumothorax, the median duration of port function was 20 days (range 1–391), and the median duration of port implantation was 391 days (range 6–723) [11].

Shunts have been used for the long-term management of pleural effusions, short-term complications developed in half of patients treated, long-term complications occurred in 8 of 11 dogs in one study [1]. Omentalization of the pleural space was employed in dogs with chylothorax in several case reports, but no controlled prospective studies have been performed to demonstrate clinical benefits when combined with other techniques [21,22]. A modified vascular access device was also employed to manage pleural effusion [12]. PleuralPort^TM^ consisting of a tube and a suction hub to be inserted into the subcutaneously is used in both dogs and cats. The pleural drainage can be performed at home by aspiration through a Huber needle inserted in the port.

The PleuralPort^TM^ application in dogs and cats was described percutaneously or in open surgery during thoracotomy procedures [11]. The former has the advantage of minimal invasiveness but does not allow the assessment of the pleural space or the taking of sampling for diagnostic purposes. The latter allows good visualization of the hemithorax but is an invasive procedure [23]. The thoracoscopic application of the device combines the advantages of minimal invasiveness, pleural cavity exploration associated with image magnification, and the performance of biopsies or other surgical procedures [1]. The data of our study showed no procedure-related technical complications and were comparable to the thoracotomy drainage application. The short- and long-term complications appear to be no different than those reported in other studies. Similar to what has been described above, case 7 presented pneumothorax that led to a resolution of the condition with repeated air aspiration. In a previous report, one cat developed persistent pneumothorax after port placement and was euthanized [11]. Our patient who developed pneumothorax was suffering from chronic chylous effusion, a condition that can cause pleural fibrosis due to chronic inflammation. This can lead to the progressive constriction of the lung parenchyma and lung entrapment [24]. Pleural fibrosis can lead to the development of pneumothorax after thoracocentesis [25]. The procedure described allows PleuralPort^TM^ insertion and biopsies execution simultaneously, it avoids the patient having to undergo two procedures under general anesthesia, and requires a single hospitalization. This reduces the stress on the dog and the costs incurred by the owner. The histopathological examination of the lesions makes it possible to obtain a diagnosis of certainty and to administrate intracavitary chemotherapy via the device in a short time. The spontaneous resolution of chylothorax was reported before in the literature [26]. The primary surgical treatment involves ligation of the thoracic duct, subtotal pericardiectomy, or a combination of both by thoracotomy or thoracoscopy [25,27,28], but recurrences may occur [29,30]. The chyle accumulation results in pleural thickening and pleural effusion-related symptoms, and intervention is necessary to improve the patient’s quality of life. The use of the pleural port for refractory chylothorax has already been described [31]. In the present case of chylothorax that relapsed after duct ligation, the resolution occurred after 1 year of regularly continued aspiration. The device does not always resolve the effusion but is a valuable aid in the management of the chronic pathological condition [11]. It is possible that the patient with chylothorax, refractory to surgical treatment has developed a condition similar to the one described and, following thoracoscopy and drainage placement, has developed pneumothorax. 

Canine malignant mesothelioma is an uncommon neoplasm associated with a poor prognosis [32], in more than 90% of cases it originates from the mesothelial cells of the pleural serosa, very rarely from the peritoneal serosa, pericardial serosa, and the tunica vaginalis of the testis [33]. The final diagnosis is through histology, so performing surgical biopsies is a key step in the diagnostic process [34]. The therapy of choice is still uncertain; chemotherapy increases patients’ survival time and reduces pleural effusion [35].

The use of intracavitary chemotherapy was previously described [19,33,36]. The five dogs affected by mesothelioma received four administrations of intrapleural cisplatin 50 mg/m^2^ every 3 weeks, as previously described [19]. The PleuralPort^TM^ device allowed repeated administration through the port in addition to drainage. Intracavitary chemotherapy was associated with a longer survival time and a reduction in pleural effusion due also to an induced chemical pleurodesis. Cisplatin concentration is higher in neoplastic tissue after intrapleural administration, and it could be responsible for long-term palliation [19]. Cisplatin is infrequently used in veterinary medicine due to its nephrotoxic effects, but intracavitary administration is better tolerated and reduces the possibility of this occurring [35]. In our study, none of the dogs that received intrapleural cisplatin showed signs of renal toxicity. Our dog receiving chemotherapy had a median survival time of 5 months. A recent study described the use of port access for intracavitary chemotherapy similar to the present study [20]. The use of a subcutaneous access port for drug administration has several advantages: avoiding repeated thoracocentesis, iatrogenic injuries or infections, and repeated sedation. In our cases, chemotherapy was well tolerated, and improved the quality of life and yielded owner satisfaction. In a recent study, Moberg investigated the treatment and the outcome of 34 dogs with malignant mesothelioma. Twenty dogs had subcutaneous port access, and twenty-five patients received intracavitary and/or intravenous chemotherapy [20]. The median survival time in dogs that received chemotherapy was 234 days and 29 in untreated dogs. Another study by Lajoinie included 40 dogs, 27 of which received chemotherapy and 13 not [35]. The resolution of the effusion after the first administration of chemotherapy was positively correlated with survival in the treated group of dogs. In both studies, chemotherapy significantly increased the median survival time, but it was not possible to perform a multivariate analysis to identify a chemotherapy protocol correlated with a longer survival time. In human medicine, the response rate is better with the combination of multiple chemotherapy agents than with the use of a single agent [37]. In our study, all dogs with a histopathological diagnosis of pleural mesothelioma were treated with the same chemotherapy protocol. The combination of piroxicam, a non-steroidal anti-inflammatory, with intracavitary cisplatin has recently been described. The preliminary results suggest that piroxicam may potentiate the effects of classical chemotherapy [33]. A first-line protocol for the treatment of malignant mesothelioma in dogs has not yet been identified, further studies and multivariate analyses are needed. Complications associated with chest tubes are discharge around the tube insertion site, accidental removal, tube blockage, accidental tube displacement, and air leaking with subcutaneous emphysema [38]. The PleuralPort^TM^ was in place for 4–5 months in our cases and in one for 1 year, and despite the long-term permanence no local reaction, infection, displacement, or other implant complications were noted. The tube and the subcutaneous port appeared relatively inert and well tolerated despite the home management. The vascular access port devices have a smaller diameter than chest tubes and appear to induce less local irritation and be less painful. In addition, subcutaneous implantation appears to reduce the risk of ascending infections [12].

Implant obstruction is quite a common complication in patients with PleuralPort^TM^ devices, and Brooks described an obstruction in 3 of 11 patients [11]. One dog died because of a complication for the accumulation of fluid in the pleural space. In the present study, in case 6, the port became obstructed after 45 days, but a one-time flushing with sterile isotonic saline solution restored the patency of the tube.

The study has some limitations. The first is the small number of subjects included and the heterogeneity of the diseases they suffered from. Further studies should be conducted to compare different modes of application of the PleuralPort^TM^ and assess the pain scores of patients in the post-operative period. The aim of the present study was to demonstrate the thoracoscopic applicability of the device. The results affirm that this minimally invasive application through thoracoscopy makes insertion feasible and adds the advantages of allowing for biopsy procedures as well as exploration of the pleural space.

## 5. Conclusions

Thoracoscopy allows for the application of the PleuralPort^TM^ device, the exploration of the pleural cavity, and the execution of biopsies of the visible lesions. The device allows the pleural cavity to be drained without performing repeated thoracocenteses, avoiding the associated complications, and allows a histopathological diagnosis to be made if samples are taken.

## Figures and Tables

**Table 1:** The table shows the data regarding signalment, number of devices applied, surgical time, diagnosis, complications and follow-up of the seven dogs included.

Case	Signalment	PleuralPort	Surgical Time	Diagnosis	Complications	Outcome
1	11 yo FN Yorkshire	2	55 min	Malignant mesothelioma	None	Euthanasia after 4 months
2	8 yo MN German Shepherd	1	80 min	Malignant mesothelioma	None	Euthanasia after 5 months
3	8 yo FN Italian Greyhound	1	35 min	Malignant mesothelioma	None	Euthanasia after 6 months
4	10 yo FS Doberman Pincher	1	45 min	Malignant mesothelioma	None	Euthanasia after 5 months
5	14 yo ME mixed breed	1	55 min	Malignant mesothelioma	None	Euthanasia after 5 months
6	15 yo FN Yorkshire	1	45 min	Pleural carcinomatosis with pulmonary metastasis and neoplastic pleural effusion	Port obstruction after 45 days	Euthanasia after 5 months
7	13 yo ME Dachshund	1	45 min	Chronic chylothorax	Pneumothorax immediately after port placement and for 12 h	Resolution after 355 days

## Data Availability

Data are contained within the article.
